# Waterlogging accelerates the loss of soil organic carbon from abandoned paddy fields in the hilly terrain in subtropical China

**DOI:** 10.1038/s41598-017-14820-z

**Published:** 2017-11-06

**Authors:** Xiao Li Xie, Wei Wang, Wen Wen Tian, Ke Jun Xie

**Affiliations:** 1 Key Laboratory of Agro-ecological Processes in Subtropical Region, Institute of Subtropical Agriculture, Chinese Academy of Sciences, Changsha, 410125 Hunan China; 2 Hunan Agricultural Resources and Environmental Protection Management Station, Changsha, 410008 Hunan China

## Abstract

Paddy soils have been widely recognized as important carbon sinks. However, paddy field abandonment is increasing in the hilly area in subtropical China. Soil waterlogging and weed burning are common practices in abandoned paddy fields, which could affect vegetation cover and carbon sequestration. An rice cultivation experiment was ceased in 2006, and four new treatments were applied as waterlogging (W), drainage (D), waterlogging combined with burning (WB), and drainage combined with burning (DB). Waterlogging altered the vegetation cover and caused an associated change in biomass. *Paspalum paspaloides*, *Murdannia triquetra*, and *Bidens frondosa* dominated W and WB plots, and *Microstegium vimineum* and *Bidens frondosa* dominated D and DB plots. Abandonment of paddy fields led to a rapid decrease in soil organic carbon (SOC), and waterlogging accelerates SOC loss which should be attributed mainly to alteration of the vegetation cover. Six years’ rice cultivation increased SOC content by 13.5% (2.4 g kg^−1^) on average. In contrast, six years’ abandonment reduced SOC content by 14.5% (3.0 g kg^−1^) on average. Decline rate of SOC was 0.38, 0.64, 0.30, and 0.65 g kg^−1^ a^−1^ for D, W, DB, and WB, respectively. Such results indicate a significant risk of SOC loss from abandoned paddy fields.

## Introduction

China is the largest rice producer in the world, with rice cultivation area of about 30 million ha in 2015, accounting for 18% of crop planting area across the country. Most paddy fields in China are located in subtropical areas with plenty of rainfall and radiation energy. Based on 404 observations across mainland China, Pan *et al*. have estimated that the average SOC change rate in topsoil (0~20 cm) is 0.110 ± 0.244 g kg^−1^ a^−1^ for rice paddy between 1985 and 2006^1^. Wu analyzed soils sampled from some 2,700 sites under contrasting land uses from the 1980s to the 2000s, and concluded that rice paddy in subtropical China accumulated soil organic carbon (SOC) faster than other arable land and some woodland^2^. Rice cultivation can increase SOC even under unfertilized conditions. Using a long-term fertilizer experiment located in subtropical China, Shang *et al*. estimated that topsoil SOC sequestration rate in double rice paddy (two rice crops per year) was 0.96 t C ha^−1^ a^−1^ for the control and 1.01~1.43 t C ha^−1^ a^−1^ for the fertilizer plots^3^. It is estimated that SOC saturation value of paddy field in south China is 26~28 g kg^−1^ ^4^. With an increasing rice productivity, paddy soils in subtropical China still have the potential to increase SOC.

Land use change is a major driving factor for the balance of SOC stocks and the global carbon cycle^5^. Land use change directly alters vegetation cover and microenvironment, which further changes the quantity and quality of organic matter in soil that derives from plants as well as the decomposition rate of soil organic matter, resulting in changes in soil carbon pool^6–9^. In hilly regions, paddy fields are usually small and fragmentary, so the efficiency is low due to the difficulty to promote mechanical agricultural production. At the same time, China is undergoing a stage of rapid urbanization and many farmers gradually abandon such cropland and migrate to cities for higher-income jobs. Therefore, paddy field abandonment is increasing in hilly areas. Changes in SOC upon land use change may occur due to changes in carbon input and/or carbon decomposition rates^5,10^. In paddy ecosystems, substantial accumulation of SOC is attributed to high input of plant residues due to increasing rice production in recent decades and retarded decomposition under anaerobic conditions^11^. Increasing number of studies have reported that abandoned agricultural land will be occupied by natural vegetation and lead to SOC accumulation^5,8,12,13^. However, these studies focused on abandoned dryland. To our knowledge, change of SOC content in abandoned paddy fields in the subtropical area has been rarely reported. Shift from paddy fields to uncultivated land will result in dramatic change in soil environment. Field management, such as tillage, fertilization, irrigation, *etc*. will be ceased, and vegetation will be changed from crops to native vegetation. All these changes will lead to alteration in organic carbon input and decomposition, as well as other nutrient transformations^14,15^.

Soil waterlogging and weed burning are common practices in abandoned paddy fields. In hilly regions, rice paddies are generally located in low-lying areas, and some abandoned paddy fields become waterlogged area due to poor drainage. Abandoned paddy fields are occupied by natural vegetation. And weed burning in winter is a common practice in abandoned paddy fields to avoid woody plant colonization, in case of reutilization of such land for crop land in the near future. Soil waterlogging and weed burning may alter vegetation cover in abandoned paddy fields and cause an associated change in belowground roots, which will affect organic carbon input. At the same time, soil water content will exert an impact on decomposition and mineralization of soil organic matter, thus further influencing the accumulation of soil organic carbon^11,16,17^. Additionally, the weed burning of aboveground weed residues may also influence the accumulation of soil organic carbon.

In this study, we established an abandoned rice paddy experiment with four treatments, including waterlogging, drainage, waterlogging combined with burning, and drainage combined with burning. The objective was to examine the effects of soil waterlogging and weed burning on vegetation and SOC content in abandoned paddy fields.

## Results

### Cover Plant Composition and Biomass

Vegetation composition and Importance Value under different treatments are listed in Table 1. *Paspalum paspaloides*, *Murdannia triquetra*, *Bidens frondosa*, *Microstegium vimineum*, *Nepeta fordii*, *Polygonum flaccidum*, *Cyperus iria*, and *Alternanthera philoxeroides* are widespread species in the experimental plots. Waterlogging altered the vegetation composition. The dominant species were different between W and B as well as between WB and DB. *Paspalum paspaloides*, *Murdannia triquetra*, and *Bidens frondosa* dominated W and WB plots. *Microstegium vimineum* and *Bidens frondosa* dominated D and DB plots. *Paspalum paspaloides* and *Murdannia triquetra* are perennial hygrophyte; *Microstegium vimineum* is annual mesophyte; and *Bidens frondosa* is annual tropophyte. Vegetation compositions are similar between W and WB as well as between D and DB, indicating that weed burning had little effect on vegetation composition.

**Table 1:** Importance Value of the major plant species under different treatments in abandoned paddy fields in 2012 and 2013.

Species	2012				2013			
	D	W	DB	WB	D	W	DB	WB
*Paspalum paspaloides*	0.02	0.22	0.04	0.30	—	0.19	0.03	0.14
*Murdannia triquetra*	0.04	0.09	0.05	0.14	0.04	0.09	0.05	0.21
*Bidens frondosa*	0.11	0.22	0.17	0.10	0.12	0.22	0.10	0.13
*Microstegium vimineum*	0.35	0.15	0.33	0.04	0.34	0.15	0.33	0.04
*Nepeta fordii*	0.06	0.06	0.09	0.08	0.05	0.06	0.05	0.07
*Polygonum flaccidum*	0.05	0.06	0.08	0.11	0.06	0.07	0.05	0.07
*Cyperus iria*	0.03	0.05	0.04	0.05	0.03	0.04	0.04	0.05
*Alternanthera philoxeroides*	0.05	0.09	0.06	—	0.03	0.10	0.03	—
*Echinochloa crusgalli*	—	—	—	0.03	—	—	0.02	0.04
*Heleocharis yokoscensis*	0.02	0.03	—	—	—	0.03	0.01	0.04
*Scirpus wallichii*	—	—	—	0.03	—	0.04	0.01	0.05
*Polygonum perfoliatum*	0.07	—	0.07	—	0.08	—	0.04	—
*Imperata cylindrica*	0.05	—	—	—	0.04	—	—	—
*Miscanthus sinensis*	0.04	—	—	—	0.04	—	—	—
*Lactuca graciliflora*	0.04	—	—	—	—	—	0.05	—
*Typha latifolia*	—	—	—	—	—	—	0.01	—
*Lindernia angustifolia*	—	—	—	—	0.01	—	—	0.03
*Lindernia nummularifolia*	—	—	—	—	0.01	—	0.01	0.01

—, not found; D, drainage; W, waterlogging; DB, drainage combined with burning; WB, waterlogging combined with burning

**Figure 1:**
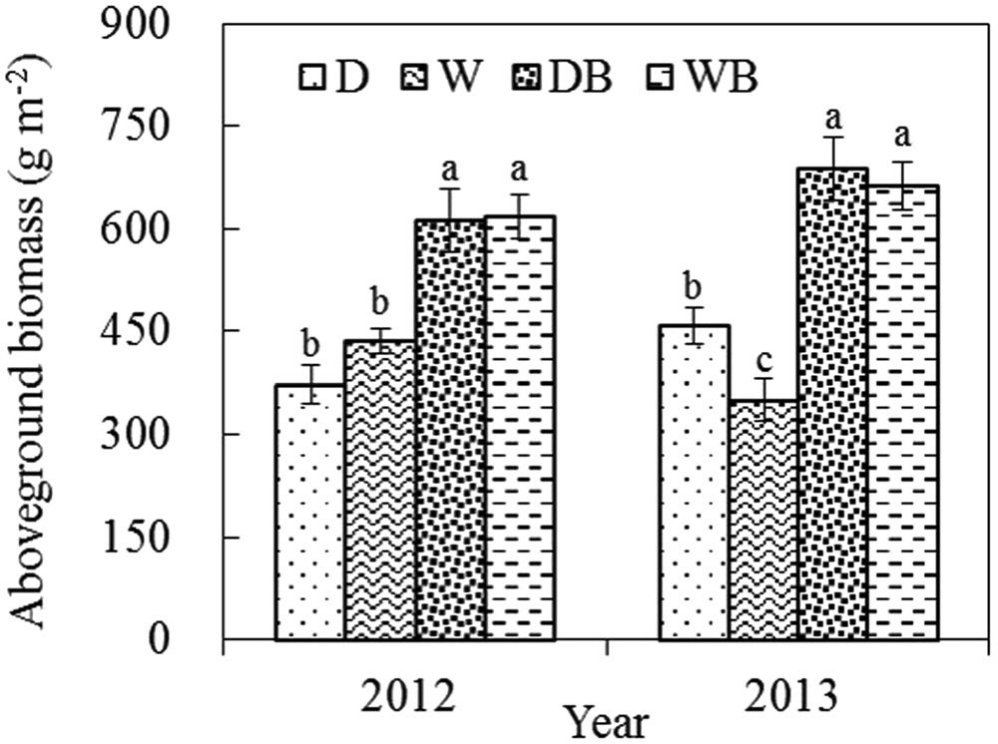
Aboveground biomass of weed under different treatments (mean ± SE, *n* = 5). D, drainage; W, waterlogging; DB, drainage combined with burning; WB, waterlogging combined with burning. Different letters indicate significant differences among treatments in the same year (P < 0.05).

The aboveground biomass varied among different treatments from 372 g m^−2^ to 688 g m^−2^ (Fig. 1). Waterlogging had little effect on aboveground biomass. Weed burning increased aboveground biomass, irrespective of waterlogging. Compared with D, DB increased aboveground biomass by 57.3% on average in 2012 and 2013. Compared with W, WB increased aboveground biomass by 65.7% on average in 2012 and 2013.

**Figure 2:**
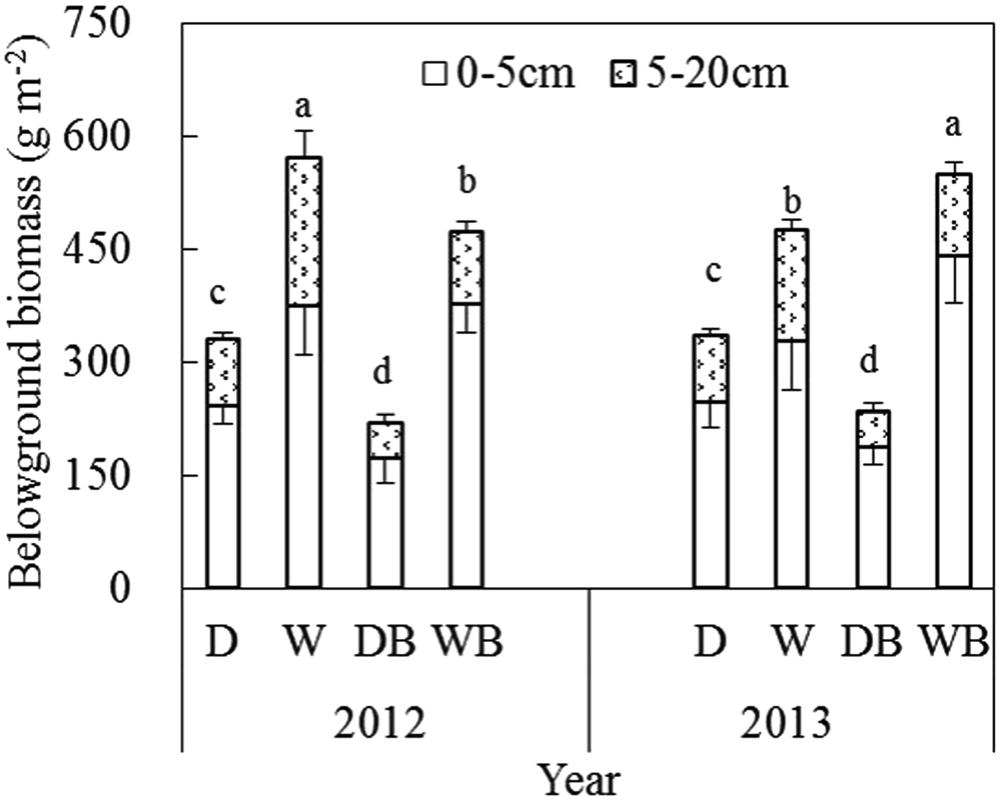
Vertical distribution of weed root biomass in the soil layers of 0~5 cm and 5~20 cm under different treatments (mean ± SE, *n* = 5). D, drainage; W, waterlogging; DB, drainage combined with burning; WB, waterlogging combined with burning. Different letters indicate significant differences of root biomass in the soil layers of 0~20 cm among treatments in the same year (P < 0.05).

The root biomass in the soil layer of 0~20 cm varied among different treatments from 219 g m^−2^ to 549 g m^−2^ (Fig. 2). The root biomass was mainly distributed in the surface soil layer of 0~5 cm, accounting for 66~80% of that in the soil layer of 0~20 cm. Waterlogging increased belowground biomass. Compared with D, W increased belowground biomass by 58% on average in 2012 and 2013. Compared with DB, WB increased belowground biomass by 125% on average in 2012 and 2013. Weed burning tended to decrease belowground biomass. Compared with D, DB decreased belowground biomass by 32% on average in 2012 and 2013. Compared with W, WB decreased belowground biomass by 2% on average in 2012 and 2013.

### Soil organic carbon

**Figure 3:**
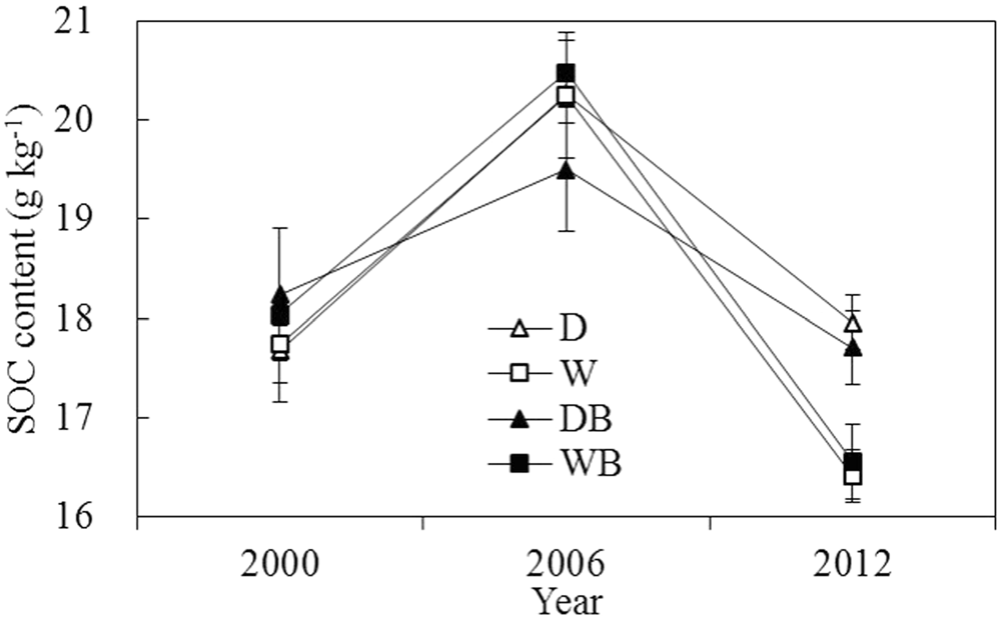
Change of SOC content during cultivation stage (2000~2006) and abandonment stage (2006~2012) (mean ± SE, n = 5). D, drainage; W, waterlogging; DB, drainage combined with burning; WB, waterlogging combined with burning.

In the six years before paddy field abandonment, rice cultivation increased SOC by 13.5% (2.4 g kg^−1^) on average in the twenty plots (Fig. 3). In contrast, SOC contents in every plots decreased after the field abandonment for six years. From 2006 to 2012, the SOC content decreased by 14.5% (3.0 g kg^−1^) on average in the twenty plots, which indicated that organic carbon input into the soil was far lower than SOC mineralization in the abandoned rice paddies. Weed burning had little effect on SOC content. In contrast with drainage, waterlogging accelerated SOC loss. SOC contents under W and WB were significantly lower than those under D and DB (P < 0.05). Decline rate of SOC was 0.38, 0.64, 0.30, and 0.65 g kg^−1^ a^−1^ for D, W, DB, and WB, respectively.

## Discussion

Paddy soils have been widely recognized as important carbon sinks^1,2,18,19^. In the present study, SOC content increased by 0.4 g kg^−1^ a^−1^ in the field of double rice cultivation for six years. In striking contrast, SOC content decreased by 0.5 g kg^−1^ a^−1^ following an abandonment for six years. During the stage of rice cultivation, around 4,000~5,000 kg ha^−1^ rice residues (root and stubble) and 2,000~3,000 kg ha^−1^ weed biomass (weed growing in fallow season) were incorporated into soil every year according to field observation. Also, a^14^C labeling study showed that a considerable amount of rice root exudation of photosynthesis-derived carbon (account for 8.0~19.3% of rice biomass carbon) was incorporated into SOC pool^20^. During the stage of abandonment, weed root biomass ranged from 2,500~6,000 kg ha^−1^ (Fig. 2). Obviously, organic carbon input in abandoned paddy soil was much lower than that in cultivated paddy soil. On the other hand, flooded conditions restricted decomposition of fresh organic materials^11,16,17^. The soil flooding period was relatively shorter in the stage of abandonment than that in the stage of rice cultivation, leading to the decomposition of more organic materials in abandoned paddy fields.

Similarly, some researchers reported that land use change from paddy rice cultivation to grassland, woodland, and upland crop cultivation caused SOC loss^21–23^. It has been widely recognized that land use changes from forest or grassland to crop fields lead to SOC loss, and the reverse processes lead to SOC accumulation^5,8,12,13^. However, rice paddy ecosystems are quite distinctive in SOC accumulation compared with other ecosystems in subtropical China^2^. In contrast, a study conducted in tropical Brazil showed that paddy fields loose much SOC when compared to native vegetation in the tropics^24^. That was because SOC content of the tropical forest soil (30~63 g kg^−1^) was way higher than that of the paddy field soil, even higher than SOC saturation content of the paddy field soil (26~28 g kg^−1^)^4,24^. The results suggest that whether SOC storage shows a positive or negative change depends on the capacity of an ecosystem to accumulate SOC when land use change occurs.

In the present study, waterlogging accelerated SOC loss, whereas weed burning had little effect on SOC loss. Possible reasons are ascribed as follows. First, waterlogging greatly changed vegetation composition (Table 1). The dominant plants under waterlogging conditions (W and WB) were perennial hygrophyte. In contrast, the dominant plants under drainage conditions (D and DB) were annual tropophyte. Although belowground biomass is higher under waterlogging conditions, the belowground biomass is mainly consisted of roots from perennial plants. So, it is not sure whether belowground production per year is higher under waterlogging conditions. Besides, much root from perennial plants under waterlogging conditions may slow down the regeneration of root, and consequently reduce the input of dead roots into soil. Second, weed burning increased aboveground biomass, but tended to decrease belowground biomass, the integrated effects of which played down the impact of burning on SOC.

**Figure 4:**
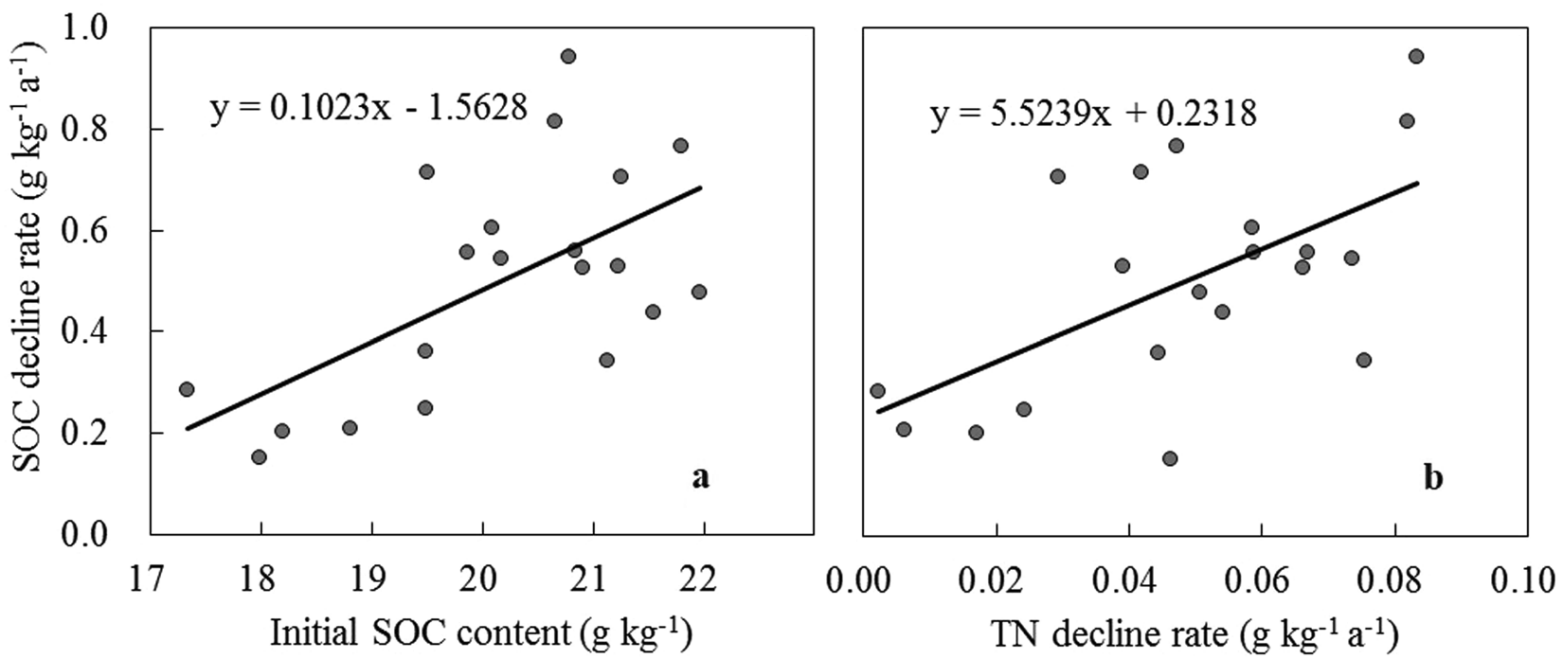
Relationship between SOC decline rate and initial SOC content (a, *r* = 0.697, P < 0.01), and relationship between SOC decline rate and TN decline rate (b, *r* = 0.590, P < 0.01).

Correlation analysis showed that SOC decline rate was positively correlated with the initial SOC content (SOC content in 2006) (*r* = 0.697, P < 0.01, Fig. 4a), indicating higher SOC decline rate for soils with higher SOC content. SOC decline rate was positively correlated with the TN decline rate (*r* = 0.590, P < 0.01, Fig. 4b), indicating a risk of nitrogen loss with decreasing of SOC. In subtropical China, rice paddy ecosystems have high SOC content, and abandonment of rice paddy fields tends to cause SOC loss accompanied by soil nitrogen loss. At present, China is still facing the challenge to feed the growing population. It is recommended that abandoned rice paddy fields be reutilized to avoid soil carbon and nitrogen loss and to ensure food safety.

## Materials and Methods

### Field trial

The investigation was conducted at Taoyuan Station of Agro-ecology Research in Taoyuan County, Hunan Province (28°55′N, 111°27′E). The region is characterized by a subtropical humid monsoon climate, with an annual average of air temperature, precipitation, sunshine, and frost-free period of 16.5 °C, 1,448 mm, 1,513 h, and 283 days, respectively. The region is featured with low hilly areas, with relative height less than 100 m. Gentle slope lands (6°~15°) account for 63% of total area. Many small and fragmented farmlands, accounting for 14%, are distributed on valley floor, the slope of which is generally smaller than 6°. The soil is developed from Quaternary red clay.

**Figure 5:**
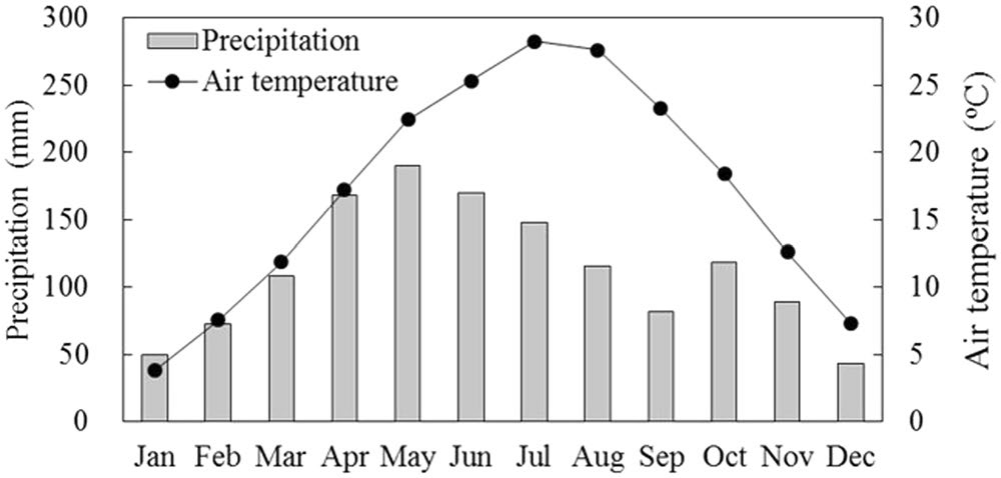
Monthly average of precipitation and air temperature from 2007 to 2012.

A double rice (two rice crops per year) field experiment was established in randomized block design with five fertilization treatments and four replicates for each treatment in 1994. The soil has been subjected to paddy farming for more than 300 years. It was flooded rice cultivation with tillage for every cropping season. The fertilization treatments were NPK, NK, NP, PK, and control (N, Nitrogen; P, phosphorus; K, potassium). Each plot was 7.5 m × 4.5 m in size. All the plots were arranged at a plane of the same altitude and surrounded by cement walls. The plots were not used for rice cultivation any more after the late-rice harvest in 2006. The twenty plots were divided into four groups, and each group comprised five plots which used to be one replicate of NPK, NK, NP, PK, and control, respectively. The four groups were arranged with four treatments which were waterlogging (W), drainage (D), waterlogging combined with weed burning (WB), and drainage combined with weed burning (DB). The five plots in each treatment was treated as replicates. Waterlogging meant collecting rainfall without drainage. The W and WB plots were usually saturated or flooded from April to August during which there was plenty of precipitation. In contrast, there was a water outlet in the cement wall for drainage, so the D and DB plots were non-flooded. Weed burning was implemented in December every year. The plant aboveground was burned to ash. Monthly averages of precipitation and air temperature from 2007 to 2012 were presented in Fig. 5.

### Vegetation investigation

Vegetation investigation was carried out in July 2012 and July 2013. Three quadrats (1 m × 1 m) were set in each plot to investigate the height, density, coverage, and aboveground biomass for each plant. Dominant species were determined on the basis of Importance Value (IV), which was calculated as IV = (relative height + relative frequency + relative coverage)/3^25^. Three columns (20 cm length × 20 cm width × 20 cm depth) were collected in each plot to investigate the belowground biomass. Each column was split into 0~5 and 5~20 cm depths to measure the vertical distribution of weed root biomass. The samples were dried at 80 °C, and weighed to calculate their biomass.

### Measurements

Soil samples from the surface layer (0~20 cm) were collected in December in 2000, 2006, and 2012. Six soil cores were collected from each plot using augers and mixed to provide a single sample. The visible pieces of plant residues (>2 mm) were removed. Soil organic carbon (SOC) was determined by dichromate oxidation method^26^. Dichromate oxidation method has been widely used in soil investigations because of its simplicity and rapidity, although it is subject to interference by oxidizable or reducible soil constituents such as Cl^−^, Fe^2+^, and MnO_2_^26^. Soil total nitrogen (TN) was determined by the Kjeldahl method^27^. Soil samples (0~20 cm, in 2006) contained 20.1 g kg^−1^ SOC, 1.78 g kg^−1^ TN, 0.76 g kg^−1^ total P, 15.2 g kg^−1^ total K, 14.9 mg kg^−1^ Olsen P, and 58.3 mg kg^−1^ available K in average. SOC decline rate (g kg^−1^ a^−1^) = (SOC_2012_ − SOC_2006_)/6. TN decline rate (g kg^−1^ a^−1^) = (TN_2012_ − TN_2006_)/6.

### Statistical analyses

All statistical analyses were performed with SPSS 17.0 (SPSS, Inc., USA) using ANOVA, followed by the Tukey’s multiple range test, in which P < 0.05 was considered statistically significant. The correlation analyses were performed using Pearson correlation analysis.
